# Maternal selenium-supplementation at various stages of periconception period: influence on murine blastocyst morphology and implantation status

**DOI:** 10.1186/s40781-017-0132-x

**Published:** 2017-04-02

**Authors:** Mark Anthony C. Mamon, Gliceria B. Ramos

**Affiliations:** Biology Department, College of Science, De La Salle University, Taft Avenue, Manila, 1004 Philippines

**Keywords:** Blastocyst morphometry, Periconception period, Pre-implantation loss, Post-implantation loss, Trace mineral, Selenium-supplementation

## Abstract

**Background:**

Selenium is one of the trace minerals whose deficiency is known to lead to complications of female reproduction. The identified gaps in researches regarding selenium and pregnancy include optimizing the dosage of selenium supplementation, timing of supplementation, finding the best form and type of selenium, and selenium administration combined with other antioxidants. Hence, this study was conceptualized to address one of the identified gaps, that is, to find out the best timing of selenium administration around the time of pregnancy. Specifically, this study aimed to assess the effects of maternal Selenium-supplementation, administered at various stages of periconception period, on murine blastocyst morphology, percent occurrence of good quality blastocysts, and implantation status.

**Methods:**

ICR female mice were randomly assigned into the unsupplemented group (Group I) receiving basal diet without selenium, and treatment groups given with 3.0 μg selenium-supplement per day during pregestation only (Group II), pregestation-throughout-gestation (Group III) and gestation only (Group IV). Both blastocyst morphology and implantation status were assessed.

**Results:**

The morphometric measurements of blastocysts appeared to be unaffected by selenium-supplementation at different stages of periconception. Selenium-supplementation at pregestation only (Group II) and gestation only (Group IV) produced higher percent occurrence of good quality blastocysts and lower percent pre-implantation loss than Group III. Among all the treatment groups, Group III (Selenium-supplementation during pregestation-to-gestation) yielded the lowest quality blastocysts and highest percent pre-implantation loss.

**Conclusion:**

Maternal selenium-supplementation during pregestation and gestation stages of the periconception period yielded a high percent occurrence of good quality blastocysts and pre-implantation success.

## Background

Selenium is a trace element with an important role in antioxidative protection [1–7]. It is a component of selenocysteine incorporated in diverse array of antioxidative selenoenzymes that include glutathione peroxidase and thioredoxin reductase, which reduce the excessive levels of reactive oxygen species (ROS) [1, 8–11]. At present, there are reports on reproductive and pregnancy complications as outcomes of selenium deficiency [2, 3, 12, 13]. However, there are insufficient evidences about the capability of selenium supplementation in preventing reproductive health and pregnancy disorders [11, 12]. In fact, there are only limited research trials of selenium supplementation during pregnancy [1, 11]. More intervention trials are recommended to determine the beneficial effects of selenium to pregnancy outcomes [12, 13].

One of the identified gaps among studies regarding selenium and reproductive health is optimizing the best timing of supplementation [3]. This lack of information about selenium is supported by other research studies recommending further investigation on the effects of timing of micronutrient administration on pregnancy outcomes [14, 15]. Hence, this present study addresses this gap by finding out the best timing of selenium administration during pregnancy, which refers to the different stages of periconception period.

Moreover, to respond with one of the United Nations Millennium Development Goals (MDG), that is, improving pregnancy outcome by optimizing the mother’s nutritional status [16], and to mitigate the alarming state of maternal deaths caused by pregnancy complications, this study aims to assess the effects of maternal Selenium-supplementation at varying stages of periconception period on murine blastocyst morphometric parameters, percent occurrence of good quality blastocysts, and implantation status. It is with hope that maternal selenium supplementation might be considered as one of the possible health measures in improving pregnancy outcome.

## Methods

### Chemicals and reagents

Selenium tablets, containing 200 μg of selenium yeast, were bought from one of the General Nutrition Centers (GNC) Live Well Health Stores in Metro Manila, Philippines. All other chemicals and reagents were purchased from other scientific chemical suppliers.

### Test animals and maintenance

Fifty six (56) seven-week old ICR female mice and twenty-eight 15–20 week old ICR male mice, approximately 30.0 g of weight were obtained from the stock bred of the Marine Science Institute – Natural Products Laboratory, University of the Philippines, Diliman. All mice were kept individually in standard - sized cages in the animal house of Department of Biology at De La Salle University – Manila, where they were acclimatized for two weeks maintaining at 12 h light: 12 h dark cycle with 28–30 °C ambient temperature. Once a week, all animal cages were sanitized, beddings were autoclaved and replaced, feed plates and water bottles were also washed

An adult mouse of 25.0 to 30.0 g weight consumes 3.0 to 6.0 g of food pellet per day [17]. Hence, all mice were given 6.0 g of food pellets per day. They had access to purified drinking water *ad libitum*.

The standard basal diet are in the form of food pellets that are available in the local pet markets in the Philippines. This is composed of wheat bran, soybean hull, soybean meal, corn, rice, alfalfa, full fat soybean, and yucca extract. Guaranteed analysis shows that these are composed of 18.0% crude protein, 16.0% crude fiber, 2.5% crude fat, 10.0% moisture, 10.0% ash, 0.9% calcium, and 0.7% phosphorus. Other macro- and micronutrients include the following: fructo-oligosaccharide, vitamin A acetate, vitamin B12 supplement, vitamin D3 supplement, folic acid, biotin, choline chloride, tocopheryl acetate, menadione sodium bisulfate, ascorbic acid, nicotinic acid, calcium-D-panthothenate, riboflavin-5′-phosphate sodium, thiamine hydrochloride, pyridoxine hydrochloride, calcium carbonate, potassium iodide, sodium chloride, monocalcium phosphate, ferrous sulfate, zinc oxide, manganous oxide, and copper sulfate.

The handling and maintenance of laboratory test animals adhered to the Rules, Regulations, and Guiding Principles of the Veterinary Medical Association’s safety standards of the Philippines and Philippine Association for Laboratory Animal Science.

### The experimental design

The study consisted of two phases; the assessments of blastocyst morphology (phase I) and implantation status (phase II).

After the acclimatization period, at which time the females were already sexually mature, were then randomly assigned into four groups with 14 members each. Two mice per group were used for phase I and twelve mice per group for phase II.

For the unsupplemented group (Group I), all were given with basal food pellets (6.0 g food pellet/day) without selenium supplement. For the treated groups, the timing for selenium administration were as follows: for the pregestation treatment group (Group II), females were given with basal food pellets + 3.0 μg selenium supplement per day for three weeks only before mating; for the pregestation-to-gestation treatment group (Group III), all were also given with diet similar with that of Group II for three weeks before mating, but females in phase I and phase II were continuously given with similar diet for 4 days and 16 days after mating respectively; for the gestation treatment group (Group IV), all mice were given with diet similar with Group II and III, but females in phase I and phase II were only given with the diet for 4 days and 16 days after mating respectively.

Mating was allowed to take place at the end of the third week (22^nd^ day). Each cage had 1 male with 2 females. To determine pregnancy, the females were checked for the presence of vaginal copulatory plugs from 0700 to 0800 h. Females that were found positive for vaginal plugs were placed in separate cages and their embryos were considered as 0.5 day post coitum (dpc) [7].

For phase I, embryo retrieval was performed at 4.5 dpc for blastocyst morphology assessment. For phase II, ovaries and uteri samples were obtained at 16.5 dpc for implantation success assessment.

### Treatment administration

Selenium supplementation was administered everyday via dietary route. The selenium supplement was prepared by dissolving a 200 μg selenium yeast tablet in 10 mL mineral water to yield 3.0 μg selenium in 0.15 mL solution.

It is the 0.15 ml solution that was coated into 2.0 g food pellets, which was given as initial consumption for the day to ensure that the whole supplement was consumed.

After the initial consumption, the remaining 4.0 g food pellets was supplied in full. The dose of selenium supplement in this study was computed based on the dosage used by Soudani et al. [18], which is 0.5 mg selenium/kg diet. This selenium dosage was proven to provide protection against oxidative stress and damage in rats.

### Phase I: blastocyst morphology assessment

Two female mice per group were sacrificed by cervical dislocation on 4.5 dpc. Blastocysts were recovered by flushing the uterus with M2 medium under a stereomicroscope. The recovered blastocysts were observed under an inverted microscope (Nikon Eclipse TS100) and were photographed using digital sight camera at 400x and 600x magnification.

**Fig. 1:**
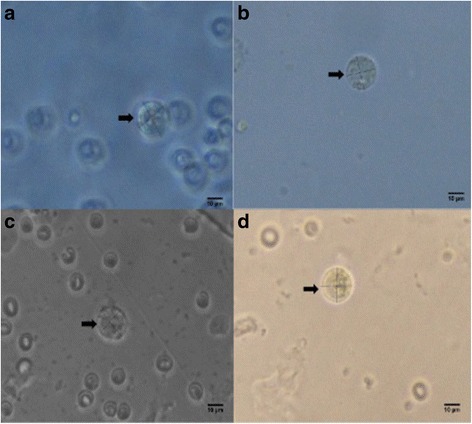
Photomicrographs of blastocysts (*arrows*) at 600x magnification being measured using the Image J software. **a** Unsupplemented group, **b** Pregestation only group, **c** Pregestation-to-gestation group, **d** Gestation only group

The images of blastocysts at 600x magnification were analyzed using the Image J software (Fig. 1). The morphometric parameters that were measured using ‘line and oval selection tools’ are as follows: mean embryo diameter (MED), mean zona pellucida diameter (MZPD), embryo area (EA), zona pellucida area (ZPA), embryo perimeter (EP), and zona pellucida perimeter (ZPP) [19, 20].

The collected embryos were classified accordingly on stages of development based on the Manual of the International Embryo Transfer Society (IETS) about ‘A procedural guide and general information for the use of embryo transfer technology emphasizing sanitary procedures’ [21]. Since all embryos were collected on 4.5 dpc, these were classified into three categories that were described as follows: compact morula with early cavitation (stage 4); early blastocyst (stage 5) described with the presence of blastocoele occupying less than half of the embryo; mid-blastocyst (stage 6) described with the presence of blastocoele occupying more than half or entirely fills the embryo [22, 23].

Blastocyst quality was scored and evaluated based also on the guidelines of the IETS Manual. The three classifications of blastocyst quality were excellent or good (score 1), fair (score 2), and poor (score 3) [21, 24]. The descriptions in each classification were determined through thresholding or binary contrast enhancement using Image J software on embryo images taken at 400x magnification.

### Phase II: implantation status assessment

On 16.5 dpc, twelve females per group were sacrificed by cervical dislocation. Uteri and ovaries were obtained, and were assessed for the number of implantations, foetuses, and corpora lutea.

Ovaries were fixed in 10% buffered formalin overnight and were brought to the Philippine Kidney Dialysis Foundation for standard histological preparations using hematoxylin and eosin stain. Ovaries were observed under an inverted microscope (Nikon Eclipse TS100) to determine the number of corpora lutea. The percent pre-implantation loss per female was calculated using this formula [25–27].

$$ \begin{array}{l}\%\ \mathrm{Pre}\hbox{-} \mathrm{implantation}\ \mathrm{loss} = \kern0.5em \left[\left(\mathrm{TCL}\ \hbox{--}\ \mathrm{TIS}\right)/\mathrm{TCL}\right]\ \mathrm{x}\ 100\\ {}\kern1.44em  where:\  TCL = total\  number\  of\  corpora\  lutea\\ {}\kern3.24em  TIS = total\  number\  of\  implantation\  sites\end{array} $$

Implantation sites were assessed by immersing the uteri into 2% sodium hydroxide (NaOH) solution until the sites become visible and clear. Implants were classified as follows: viable fetuses have distinct fetal capsule and placenta [28]; non-viable fetuses are underdeveloped and smaller than viable fetuses [27, 29] with identifiable ischemia or hemorrhage [28]; and resorptions or resorbed embryos described as small, round, black or dark brown masses, which are signs of necrosis [29]. Percent post–implantation loss per female was determined using this formula [27, 29].

$$ \begin{array}{l}\%\ \mathrm{Post}\hbox{--} \mathrm{implantation}\ \mathrm{loss} = \left[\left(\mathrm{TIS}\ \hbox{--}\ \mathrm{NVF}\right)/\mathrm{TIS}\right]\ \mathrm{x}\ 100\\ {}\kern1.32em  where:\  TIS = total\  number\  of\  implantation\  sites\\ {}\kern2.76em  NVF = number\  of\  viable\  fetuses\end{array} $$

### Statistical analysis

Statistical differences of data on blastocyst morphology among groups were analyzed by one-way analysis of variance (ANOVA) followed by post-hoc Tukey HSD test. Blastocysts were classified or scored based only on qualitative assessment. Percent pre- and post-implantation losses were analyzed using non-parametric Kruskal-Wallis test, and Mann Whitney test to determine the specific pairs of experimental groups with significant differences. Data were presented as mean ± SEM (standard error of the mean). All statistical tests were conducted using Statistical Package for the Social Sciences (SPSS) Version 22. Effects of the treatment were considered significant at *P-values < 0.05*.

## Results

### General observations

Three developmental stages of blastocysts were identified in the study. These were stage 4, characterized as compact morula with cavitation; stage 5, characterized as early blastocyst having blastocoele occupying less than half of the embryo; and stage 6, characterized as mid-blastocyst having blastocoele occupying more than half of the embryo.

### Morphometric measurements of blastocysts and occurrence of good quality blastocysts

Group III exhibited the lowest values of all the morphometric parameters (Table 1). Most of these parameters were significantly lower than Group I but not from those of treatment Groups II and IV.

**Table 1:** Morphometric parameters of mice blastocysts from all groups

Groups	*n*	Morphometric parameters					
		Mean embryo diameter (MED) μm	Mean zona pellucida diameter (MZPD) μm	Embryo area (EA) μm^2^	Zona pellucida area (ZPA) μm^2^	Emrbyo perimeter (EP) μm	Zona pellucida perimeter (ZPP) μm
Group I	*12*	21.20±0.93	25.17±1.07	376.18±33.81	509.38±41.63	68.01±3.13	79.31±3.33
Group II	*15*	19.86±0.59^a^	22.94±0.61^a^	317.94±18.97^a^	418.83±21.36^a^	62.90±1.71^a^	72.30±1.69^a^
Group III	*13*	18.60±0.58^a^	21.65±0.54^a*^	275.28±15.02^a*^	376.46±18.32^a*^	58.57±1.65^a*^	68.56±1.72^a*^
Group IV	*20*	20.21±0.43^a^	23.49±0.44^a^	301.94±12.41^a*^	422.88±15.13^a^	61.39±1.24^a^	72.70±1.27^a^

Values are given as mean±SEM. *n* = number of blastocyst

Values with the same letters in the treatment groups within column are not significantly different

Values in the treatment groups with asterisk are significantly different from the unsupplemented group

Group I (Unsupplemented group), Group II (Pregestation only group), Group III (Pregestation-to-gestation group), and Group IV (Gestation only group)

**Fig. 2:**
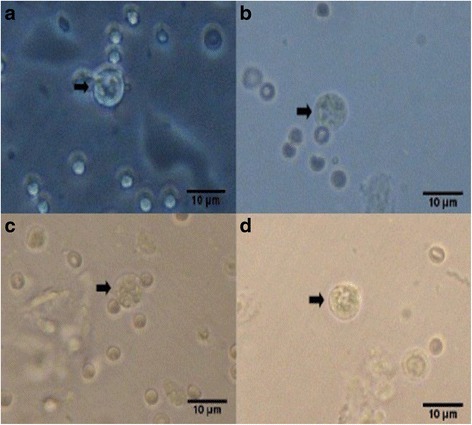
Photomicrographs of good quality blastocysts (*arrows*) at 400x magnification. **a** Unsupplemented group (Group I), **b** Pregestation only group (Group II), **c** Pregestation-to-gestation group (Group III), and **d** Gestation only group (Group IV)

**Fig. 3:**
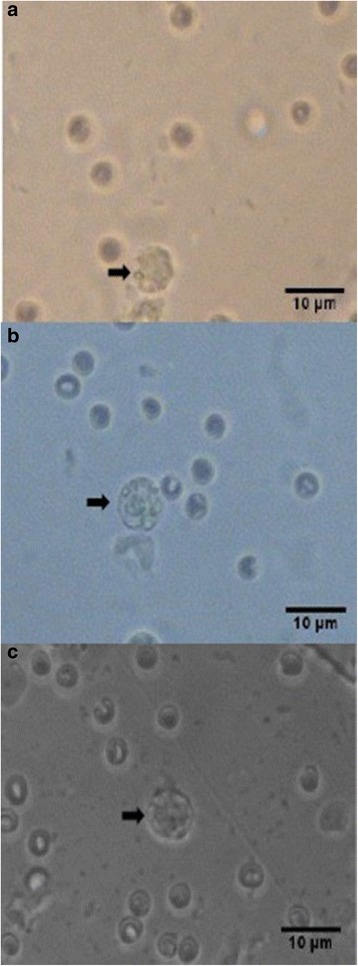
Photomicrographs of fair quality blastocysts (*arrows*) at 400x magnification. **a** Unsupplemented group (Group I), **b** Pregestation only group (Group II), and **c** Pregestation-to-gestation group (Group III)

**Fig. 4:**
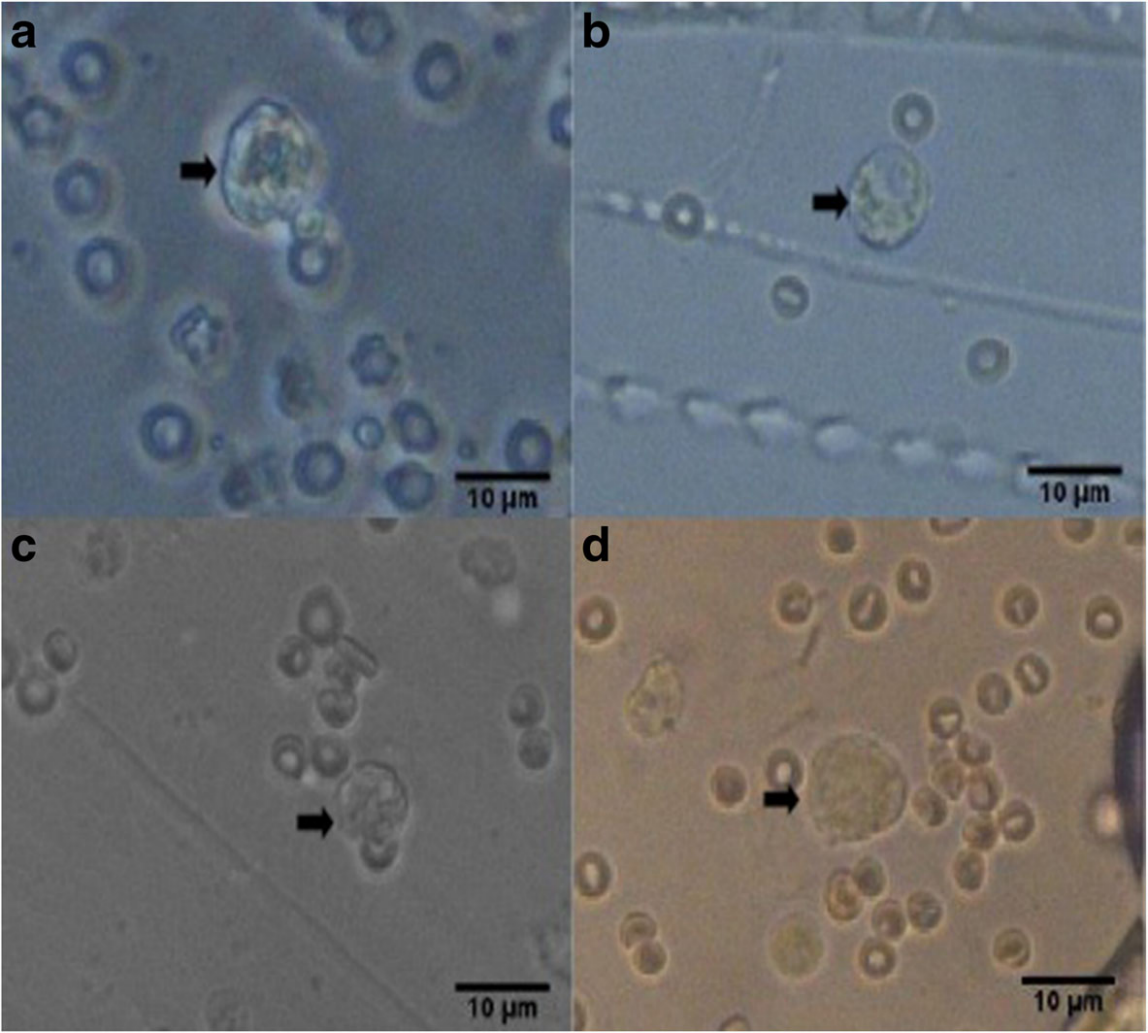
Photomicrographs of poor quality blastocysts (*arrows*) at 400x magnification. **a** Unsupplemented group (Group I), **b** Pregestation only group (Group II), **c** Pregestation-to-gestation group (Group III), and **d** Gestation only group (Group IV)

Percent occurrences of good (Fig. 2), fair (Fig. 3), and poor (Fig. 4) quality blastocysts are shown in Table 2. Group IV showed the highest percent occurrence of good quality blastocysts followed by Group II. Groups I and III were comparably low (Table 2; Figs. 2, 3, and 4).

**Table 2:** Percent occurrence of three classification of blastocyst quality following the guidelines of the IETS Manual

Groups	*n*	Actual blastocyst count (Percent Occurrence %)		
		Good (1)	Fair (2)	Poor (3)
Group I	12	4 (33.3)	6 (50)	2 (16.67)
Group II	15	11 (73.3)	3 (20)	1 (6.7)
Group III	13	5 (38.5)	4 (30.8)	4 (30.8)
Group IV	20	19 (95)	0	1 (5)

*n* = number of blastocyst

Percent in parenthesis

Group I (Unsupplemented group), Group II (Pregestation only group), Group III (Pregestation-to-gestation group), and Group IV (Gestation only group)

### Implantation losses

**Fig. 5:**
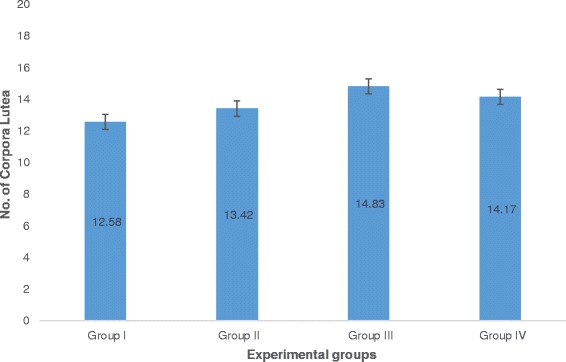
Number of corpora lutea obtained from H&E stained ovaries from all groups. Values are given as mean ± SEM. Group I (Unsupplemented group), Group II (Pregestation only group), Group III (Pregestation-to-gestation group), and Group IV (Gestation only group)

**Fig. 6:**
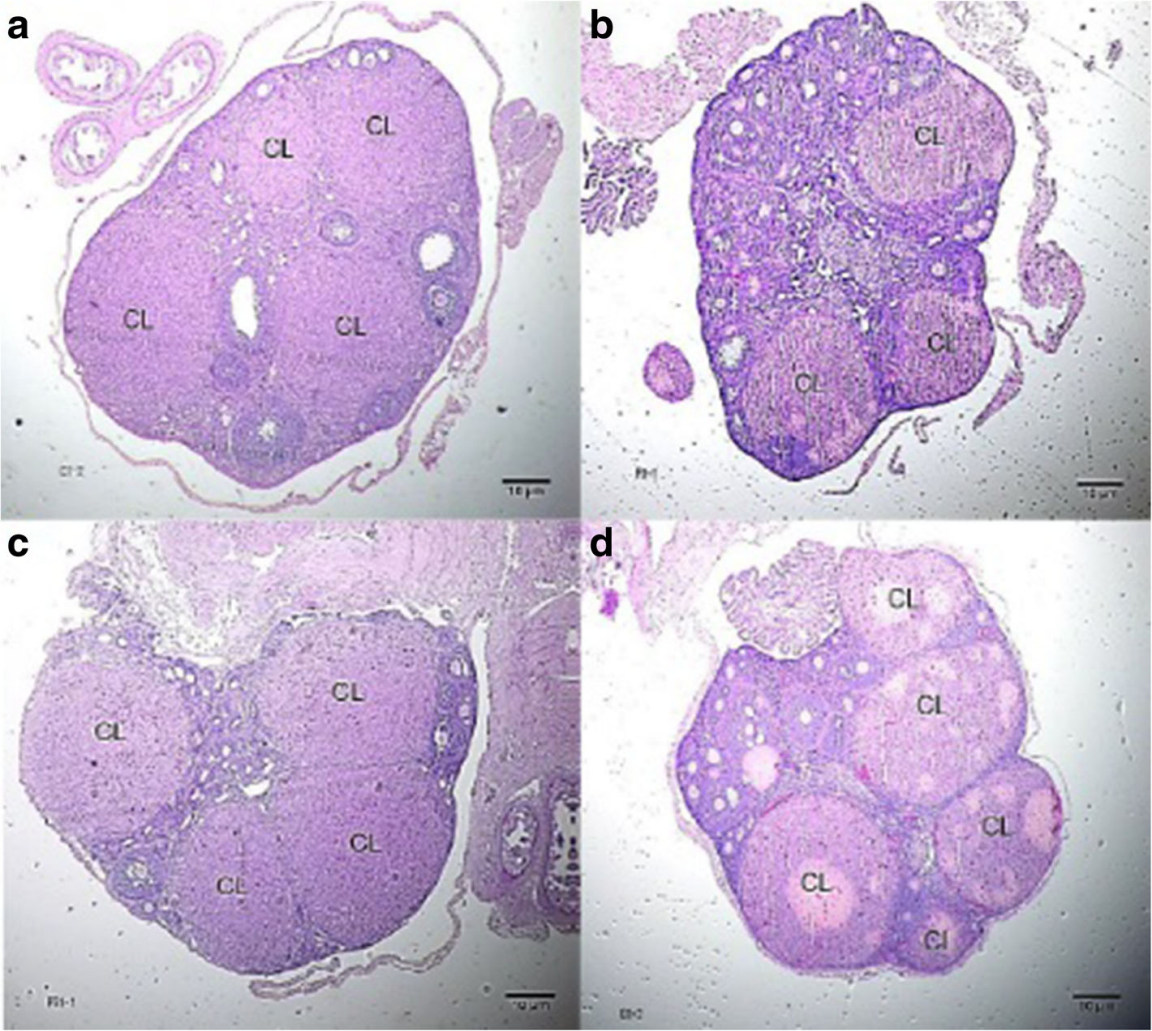
Histological cross – sections of ovaries showing the corpora lutea (CL) from all groups at 40x magnification. **a** Unsupplemented group, **b** Pregestation only group, **c** Pregestation-to-gestation group, **d** Gestation only group

The number of corpora lutea determined through hematoxylin and eosin (H&E) staining of ovaries is shown in Fig. 5 with representative histophotographs that are shown in Fig. 6. This reproductive outcome among pregnant mice is used to compute for percent pre-implantation loss.

**Fig. 7:**
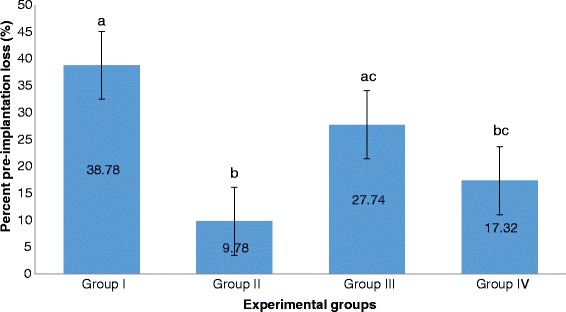
Percent pre-implantation loss among groups. Values are given as mean ± SEM. Values with the same letters are not significantly different. Group I (Unsupplemented group), Group II (Pregestation only group), Group III (Pregestation-to-gestation group), and Group IV (Gestation only group)

Group II significantly exhibited the lowest percent pre-implantation loss followed by Group IV. Both of these groups exhibited significantly lower percent pre-implantation loss than that of Group I. Group III had the highest percent pre-implantation loss among those of the treatment groups. It is significantly different from that of Group II but not from that of Group IV (Fig. 7).

**Fig. 8:**
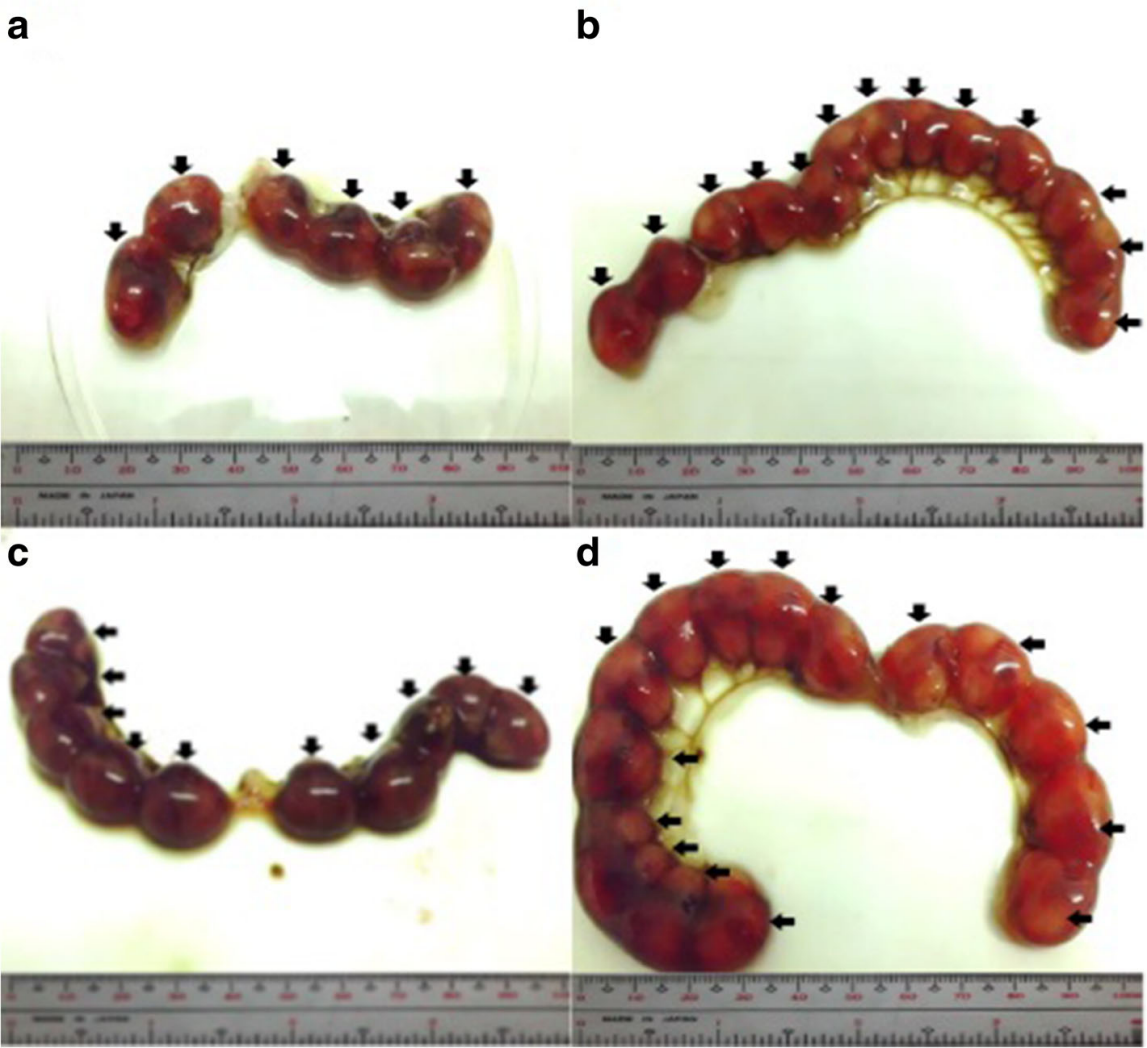
Photographs of 16.5 dpc murine uterine horns with 100% viable fetuses (*arrows*). **a** Unsupplemented group, **b** Pregestation only group, **c** Pregestation-to-gestation group, **d** Gestation only group

**Fig. 9:**
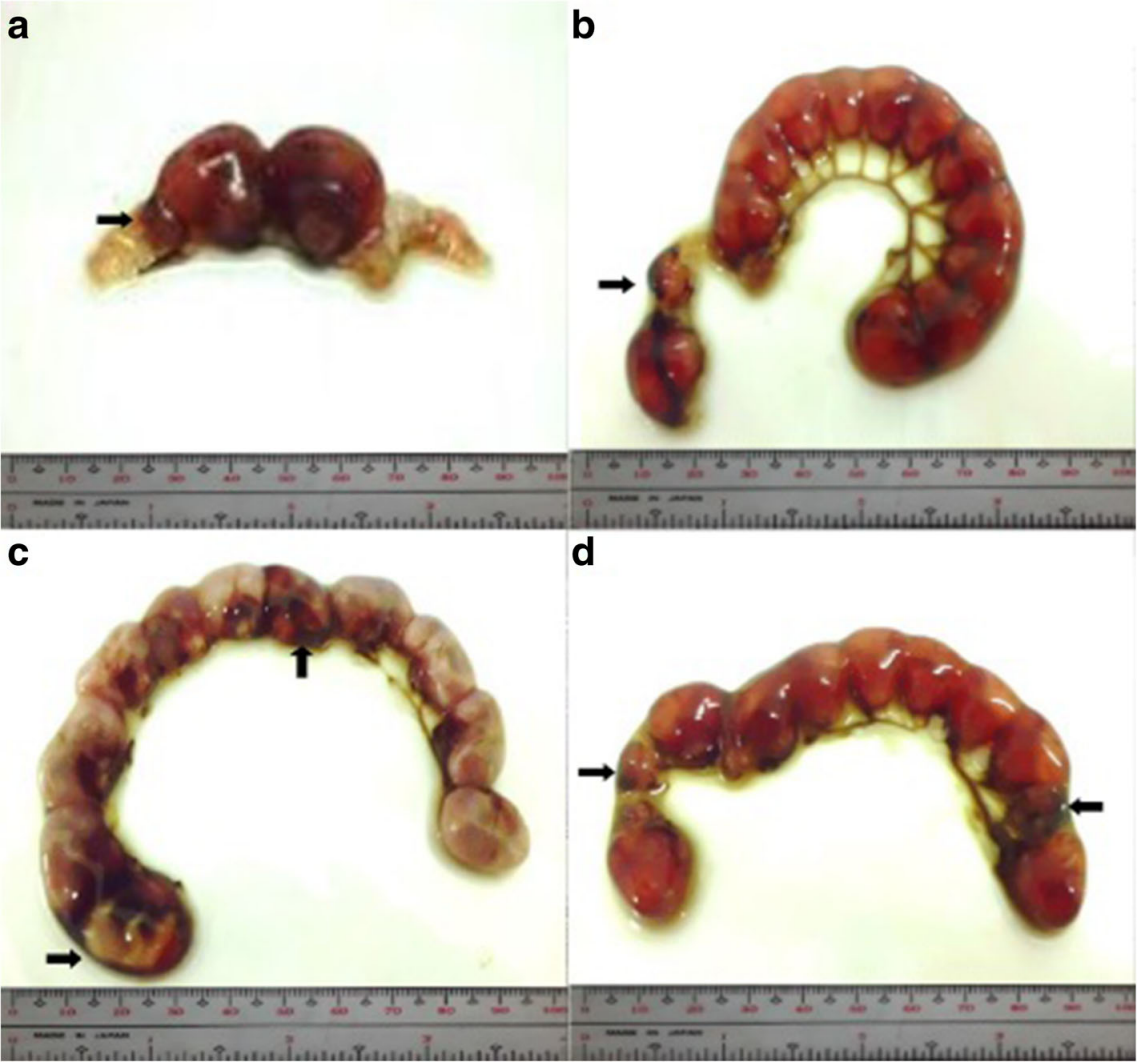
Photographs of 16.5 dpc murine uterine horns showing viable and non-viable (*arrows*) fetuses. **a** Unsupplemented group, **b** Pregestation only group, **c** Pregestation-to-gestation group, **d** Gestation only group

**Fig. 10:**
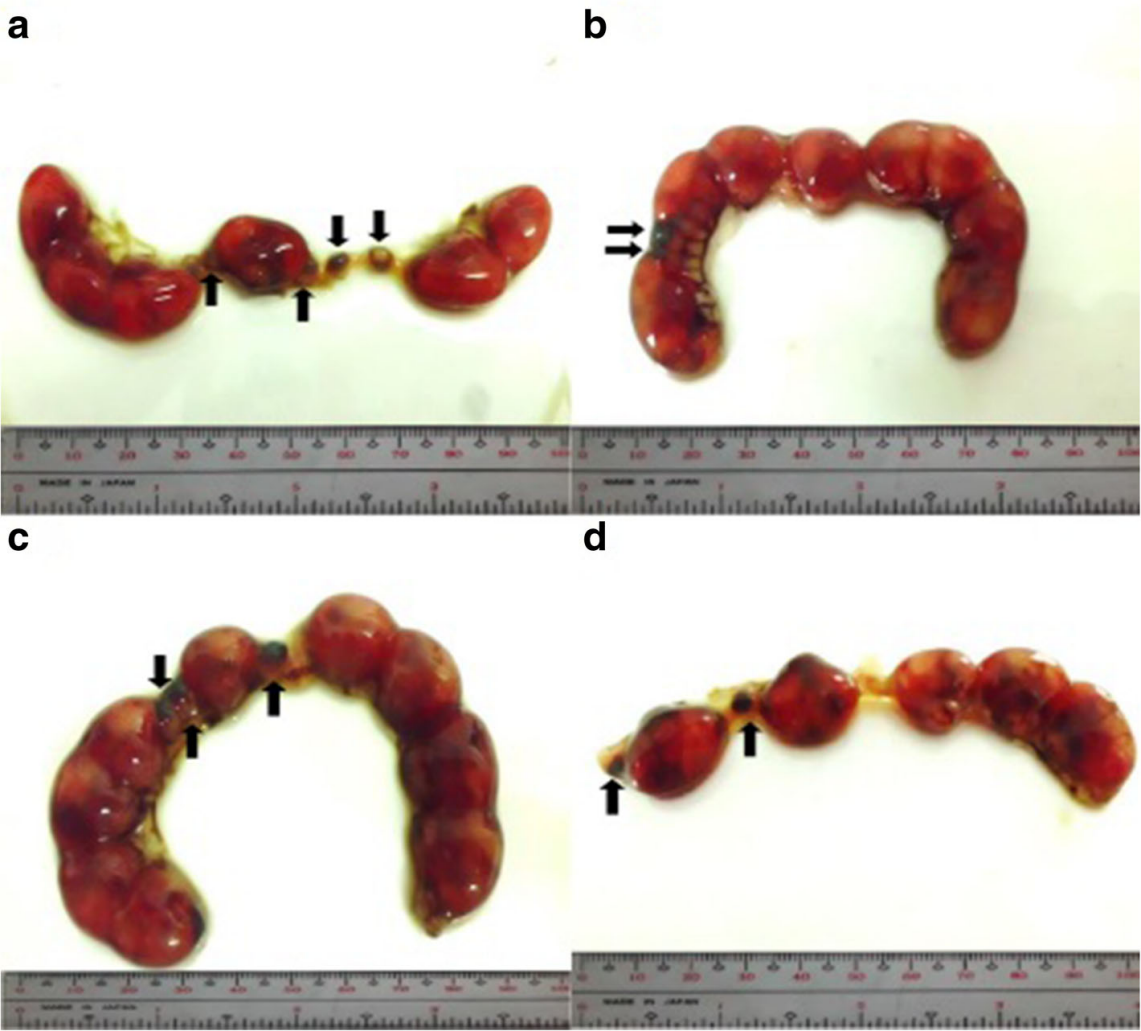
Photographs of 16.5 dpc murine uterine horns showing viable fetuses and resorptions (*arrows*). **a** Unsupplemented group, **b** Pregestation only group, **c** Pregestation-to-gestation group, **d** Gestation only group

**Fig. 11:**
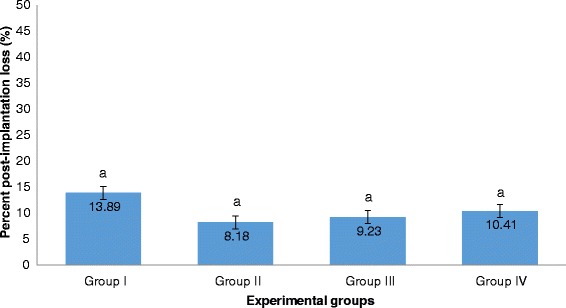
Percent post-implantation loss among groups. Values are given as mean ± SEM. Values with the same letters are not significantly different. Group I (Unsupplemented group), Group II (Pregestation only group), Group III (Pregestation-to-gestation group), and Group IV (Gestation only group)

The percent post-implantation loss that was assessed from the implantation sites in the uteri which were classified as viable fetuses, non-viable fetuses, and resorbed embryos (Figs. 8, 9, 10 and 9, respectively) showed no significant differences among all groups (Fig. 11).

## Discussion

The results of the blastocyst morphometric measurements may indicate that selenium-supplementation at varying stages of periconception has not strongly influenced these morphological parameters. It appears however, that a longer period of supplementation (25 days for Group III) may have a tendency towards yielding smaller-sized blastocysts. These selected morphometric parameters are variables that seems to be not sensitive to the effects of varying the stages of maternal selenium-supplementation.

The highest percent occurrence of good quality blastocysts, that is, the lowest number of poor quality blastocysts in Group IV may indicate that the best periconception stage for selenium-supplementation to ensure good quality blastocysts is during gestation stage. It must be taken into consideration however, that percent occurrence in Group II, is high as well relative to Group III and Group I. Thus, selenium-supplementation either during pregestation only or gestation only may have similar influence on yielding good quality blastocysts. This is further supported by the abovementioned smaller-sized embryos in Group III (Table 1), which is also the group that has the lowest percent occurrence of good quality blastocysts among all treatment groups.

Probably, the antioxidant system during these stages of periconception could have been enhanced by selenium-supplementation. The enhancement of antioxidant system can mitigate oxidative stress that increases during pregnancy because of an upsurge in maternal metabolic demand for energy production [30, 31]. Pregnancy leads to reactive oxygen species (ROS) formation that once it reached excessive levels causes detrimental effects on normal cellular functions [10, 30]. Based on many other studies, excessive ROS is damaging on normal female reproduction, specifically on embryo development [10, 32–34].

During selenium-supplementation, this micronutrient is incorporated in the selenocysteine of antioxidative selenoproteins that include isoforms of glutathione peroxidases (GPXs) and thioredoxin reductases (TrxRs), selenoprotein P and selenoprotein W [3–6, 8, 35]. Selenium-supplementation during pregestation only or gestation only could have embryoprotective capacity on the deleterious impacts and influences of ROS on the structure and function of cellular macromolecules, cell membrane and mitochondria, thus preventing abnormal and delayed embryo development [10, 36–40].

The high percent occurrence of poor quality blastocysts in Group I (Unsupplemented group) may be attributed to the lower levels of antioxidants than those of the supplemented groups. Poor quality blastocysts were reported as could be due to lower levels of antioxidants [36].

The low percent occurrence of good quality blastocyst in Group III possibly shows the toxic effects or ineffective influence of long-term selenium supplementation. Selenium has many beneficial effects to animal and human health, but this trace mineral still has reported cases of toxicity [3, 6, 41, 42], when given in high dose [43] or in long-duration [44, 45]. Taking into consideration that the selenium dose used in this study is at a level established to have a protective effect based from Soudani et al., [18], the 25 days (pregestation-to-gestation) of selenium-supplementation might have caused a certain level of toxicity.

It was reported that long-term selenium-supplementation does not result to the optimal levels and actions of selenoproteins, but led to the overexpression of these antioxidant proteins resulting further to the development of insulin resistance, hyperglycemia, and hyperinsulinemia [5, 45, 46]. Specifically, the overexpression of glutathione peroxidase 1 has undesirable effects to insulin- induced signaling pathways consequently promoting type 2 diabetes mellitus [46]. Thus, when this metabolic disorder occurs during pregnancy, ROS is produced [3], therefore risking the normal development of blastocysts.

Long-term selenium-supplementation could also affect the metabolic clearance rate of selenium, which is also influenced by the type of selenium supplement that is administered. Selenium-enriched yeast, the form of selenium supplement used in this study, is highly rich in selenomethionine (SeMet) [47, 48]. Reports show that SeMet is highly absorbed in the small intestine through methionine transporter system, and highly retained and accumulated in the organs [48–51] such as skeletal muscles, kidneys, liver, and pancreas [3, 49]. The SeMet received by female mice from selenium-enriched yeast in this study, could have lowered the excretion or the metabolic clearance rate of selenium. Further, it might have been intensified by the long-term selenium-supplementation for 25 days (pregestation-to-gestation). As a consequence, the accumulated selenium levels in the organs contributed by the organic selenium form and the selenium levels contributed by the prolonged supplementation may possibly induce embryotoxic effects to pre-implantation stage embryos, specifically to blastocysts.

The maternal selenium level damaging to blastocyst development possibly enhanced by lower metabolic clearance rate is not caused by the decline in renal hemodynamics that occurs only during the last stages of pregnancy [52, 53]. In fact, early stages of pregnancy are described with an increase in glomerular filtration rate and renal hemodynamic flow [50, 54]. Therefore, the possible lower excretion rate of selenium in female mice in this study might be an effect on the use of organic selenium supplement aggravated by long-term selenium supplementation.

The report on the reduced ovulation and implantation, and few live fetuses in rats injected intraperitoneally with high dose of sodium selenite (4.0 mg/kg body weight) [55] supports the findings of this study that selenium beyond the desired status in the maternal body could have detrimental effects to embryonic development.

Pre-implantation status was also evidently supported by selenium-supplementation in Group II. Considering that Group II is not significantly different from that of Group IV, selenium-supplementation in these periconception stages could possibly lower percent pre-implantation loss. These results in pre-implantation status of Group II and Group IV are supported by the incurred high percent occurrence of good quality blastocysts (Table 2) in these two periconception stages. Blastocysts with good quality have higher competency for successful implantation [23, 56] which is the primary determinant for successful pregnancy [57].

Aside from blastocyst quality, successful implantation depends on the endometrial receptivity to the blastocyst [56, 58, 59]. Group II having the lowest percent pre-implantation loss among groups, suggests that selenium-supplementation during pregestation only could have prepared the uterine endometrium for implantation. Since pregnancy results to a decline in glutathione peroxidase (GPx) activity [1, 3, 13], selenium-supplementation during this periconception stage could increase the levels of glutathione peroxidase before and during the early onset of pregnancy. Specifically, the isoform of GPx involved in blastocyst implantation is glutathione peroxidase 3 (GPx3,) which was upregulated for human endometrial receptivity [58], and was abnormally expressed among women who experienced implantation failure [59]. Thus, selenium-supplementation during this stage can possibly prepare and improve blastocyst implantation by enhancing the level and activity of GPx3. This selenoprotein prepares the endometrium for implantation by protecting the uterine cells and embryos from excessive ROS such as hydrogen peroxides and organic hydroxyperoxides [60].

Aside from GPxs, selenium-supplementation at pregestation stage and gestation stage can enhance the levels and activities of thioredoxin reductases (TrxRs), which are selenoproteins involved in reducing and maintaining the levels of small antioxidative protein known as thioredoxin [3, 6, 10]. This reduced state protein makes developing embryos resistant from the damages of oxidative stress [10] that in turn could promote association of blastocysts with the endometrial lining. The favorable results of selenium-supplementation during pregestation was supported by Kind [61], which states that since poor macro- and micronutrient status of the mother before conception has tremendous impact to the establishment of pregnancy, supplementation of these nutrients before pregnancy can prepare the maternal body in supporting normal embryonic development.

The high percent pre-implantation loss in Group III, though not significantly higher than that of Group IV, but significantly higher than that of Group II strengthens the findings that selenium-supplementation during pregestation-to-gestation produces the lowest quality of blastocysts among treatment groups, which may lower the chance of successful implantation.

## Conclusions

The overall findings of the present study indicate that pregestation only and gestation only are the best periconception stages for selenium-supplementation to yield high percent occurrence of good quality blastocysts and pre-implantation success.

## Abbreviations

DPC: Days post coitum
EA: Embryo area
EP: Embryo perimeter
GPx: Glutathione peroxidase
IETS: International embryo transfer society
MED: Mean embryo diameter
MZPD: Mean zona pellucida diameter
NVF: Number of viable fetuses
ROS: Reactive oxygen species
SEM: Standard error of the mean
SeMET: Selenomethionine
TCL: Total number of corpora lutea
TIS: Total number of implantation sites
TrxRs: Thioredoxin reductases
ZPA: Zona pellucida area
ZPP: Zona pellucida perimeter

## References

[1] Mistry HD, Williams PJ. The importance of antioxidant micronutrients in pregnancy. Oxid Med Cell Longev. 2011;2011:1–12. Article ID 841749. 10.1155/2011/841749 PMC3171895 21918714

[2] Palmieri C, Szarek J (2011). Effect of maternal selenium supplementation on pregnancy in humans and livestock. J Elementol.

[3] Mistry HD, Pipkin FB, Redman CWG, Poston L (2012). Selenium in reproductive health. Am J Obstet Genecol.

[4] Ostadalova I (2012). Biological effects of selenium compounds with a particular attention to the ontogenetic development. Physiol Res.

[5] Rayman MP (2012). Selenium and human health. Lancet.

[6] Mehdi Y, Hornick JL, Istasse L, Dufrasne I (2013). Selenium in the environment, metabolism, and involvement on body functions. Molecules.

[7] Ramos GB, Sia AJ, Callejas NAN, Revilla CJP, Alfonso N, Sia SG (2013). Pregestational and gestational maternal selenium – supplement: influence on ethanol – induced dysmorphogenesis in murine postimplantation embryos. Asian j exp biol sci.

[8] Tinggi U (2008). Selenium: its role as antioxidant in human health. Environ Health Prev Med.

[9] Hefnawy AEG, Perez JLT (2010). The importance of selenium and the effects of its deficiency in animal health. Small Rumin Res.

[10] Ufer C, Wang CC (2011). The roles of glutathione peroxidases during embryo development. Front Mol Neurosci.

[11] Vanderlelie J, Perkins AVA (2011). Selenium and preeclampsia. Pregnancy Hypertens.

[12] Hovdenak N, Haram K (2012). Influence of mineral and vitamin supplements on pregnancy outcome. Eur J Obstet Gynecol Reprod Biol.

[13] Pieczynska J, Grajeta H (2015). The role of selenium in human conception and pregnancy. J Trace Elem Med Biol.

[14] Cetin I, Berti C, Calabrese S (2010). Role of micronutrients in periconceptional period. Hum Reprod Update.

[15] Berti C, Biesalski HK, Gartner R, Lapillonne A, Pietrzik K, Poston L, Redman C, Koletzko B, Cetin I (2011). Micronutrients in pregnancy: current knowledge and unresolved questions. Clin Nutr.

[16] Hambidge KM, Krebs NF, Westcott JE, Garces A, Goudar SS, Kodkany BS, Pasha O, Tshefu A, Bose CL, Figueroa L, Goldenberg RL, Derman RJ, Friedman JE, Frank DN, McClure EM, Stolka K, Das A, Thomas MK, Sundberg S (2014). Preconception Trial Group, Preconception maternal nutrition: a multi-site randomized controlled trial. BMC Pregnancy Childbirth.

[17] Sahni S (2000). Guidelines for Care and Use of Animals in Scientific Research.

[18] oudani N, Amara IB, Sefi M, Boudawara T, Zeghal N. Effects of selenium on chromium (VI) – induced hepatotoxicity in adult rats. Exp Toxicol Pathol. 2011;63:541–8. 10.1016/j.etp.2010.04.005 20494564

[19] Matos FD, Rocha JC, Nogueira MFG (2014). A method using artificial neural networks to morphologically assess mouse blastocyst quality. J Anim Sci Technol.

[20] Molina I, Ibañez EL, Pertusa J, Debon A, Sanchis JVM, Pellicer A (2014). A minimally invasive methodology based on morphometric parameters for day 2 embryo quality assessment. Reprod Biomed.

[21] Bo GA, Mapletoft RJ (2013). Evaluation and classification of bovine embryos. Anim Reprod.

[22] Baczkowski T, Kurzawa R, Glabowski W (2004). Methods of embryo scoring in in vitro fertilization. Reprod Biol.

[23] Kovacic B, Vlaisavljevic V, Wu B (2012). Importance of blastocyst morphology in selection for transfer. Advances in Embryo Transfer. Biochemistry, Genetics, and Molecular Biology.

[24] Racowsky C, Vernon M, Mayer J, Ball GD, Behr B, Pomeroy KO, Wininger D, Gibbons W, Conaghan J, Stern JE (2010). Standardization of grading embryo morphology. J Assist Reprod Genet.

[25] Bindali BB, Kaliwal BB (2002). Anti – implantation effect of a carbamate fungicide Mancozeb in Albino mice. Ind Health.

[26] Ambali SF, Imana HO, Shittu M, Kawu MU, Salami SO, Ayo JO (2010). Anti–implantation effect of chlorpyrifos in Swiss albino mice. Agric Biol J N Am.

[27] Yeh J, Kim BS, Peresie J (2011). Reproductive toxic effects of cisplatin and its modulation by the antioxidant sodium 2 – mercaptoethanesulfonate (mesna) in female rats. Reprod Bio Insights.

[28] Zhao Y, Wang X, Shi W, Zhong X (2011). Anti-abortive effect of quercetin and bornyl acetate on macrophages and IL-10 in uterus of mice. Afr J Biotechnol.

[29] Yu WJ, Kim JC, Chung MK (2008). Lack of dominant lethality in mice following 1–bromopropane treatment. Mutat Res.

[30] Boskabadi H, Omran FR, Tara F, Rayman MP, Mobarhan MG, Sahebkar A, Tavallaie S, Shakeri MT, Alamdari DH, Kiani M, Razavi BS, Oladi M, Ferns G (2010). The effect of maternal selenium supplementation on pregnancy outcome and the level of oxidative stress in neonate. Iran Red Crescent Med J.

[31] Cebovic TN, Maric D, Nikolic A, Mikic AN (2013). Antioxidant status in normal pregnancy and preeclampsia upon multivitamin-mineral supplementation in the region of Vojvodina. Int J Biosci Biochem Bioinforma.

[32] Agarwal A, Gupta S, Sikka S (2006). The role of free radicals and antioxidants in reproduction. Curr Opin Obstet Gynecol.

[33] Gupta S, Agarwal A, Banerjee J, Alvarez JG (2007). The role of oxidative stress in spontaneous abortion and recurrent pregnancy loss: A systematic review. Obstet Gynecol Surv.

[34] Agarwal A, Mellado AA, Premkumar BJ, Shaman A, Gupta S (2012). The effects of oxidative stress on female reproduction: a review. Reprod Biol Endocrinol.

[35] Lu J, Holmgren A (2009). Selenoproteins. J Biol Chem.

[36] Yang HW, Hwang KJ, Kwon HC, Kim HS, Choi KW, Oh KS (1998). Detection of reactive oxygen species (ROS) and apoptosis in human fragmented embryos. Hum Reprod.

[37] Guerin P, El Mouatassim S, Menezo Y (2001). Oxidative stress and protection against reactive oxygen species in the pre-implantation embryo and its surrounding. Hum Reprod Update.

[38] Agarwal A, Allamaneni SSR (2004). Oxidants and antioxidants in human fertility. Middle East Fertil Soc J.

[39] Cebral E, Carrasco I, Vantman D, Smith R (2007). Preimplantation embryotoxicity after mouse embryo exposition to reactive oxygen species. Biocell.

[40] Sobrinho DBG, Oliveira JBA, Petersen CG, Mauri AL, Silva LF, Massaro FC, Baruffi RL, Cavagna M, Franco JG (2011). IVF/ICSI outcomes after culture of human embryos at low oxygen tension: a meta-analysis. Reprod Biol Endocrinol.

[41] Mezes M, Balogh K (2009). Prooxidant mechanisms of selenium toxicity – a review. Acta Biol Szeged.

[42] Fairweather-Tait SJ, Bao Y, Broadley MR, Collings R, Ford D, Hesketh JE, Hurst R (2011). Selenium in human health and disease. Antioxid Redox Signal.

[43] Jansen E, Viezeliene D, Beekhof P, Gremmer E, Rodovicius H, Sadauskiene I, Ivanov L (2013). Biomarkers of selenium toxicity after sub-acute exposure in mice. J Mol Biomark Diagn.

[44] Puspitasari IM, Abdulah R, Yamazaki C, Kameo S, Nakano T, Koyama H (2014). Updates on clinical studies of selenium supplementation in radiotherapy. Radiat Oncol.

[45] Stranges S, Marshall JR, Natarajan R, Donahue RP, Trevisan M, Combs GF, Cappuccio FP, Ceriello A, Reid ME (2007). Effects of long-term selenium supplementation on the incidence of type 2 diabetes. Ann Intern Med.

[46] Steinbrenner H, Speckmann B, Pinto A, Sies H (2011). High selenium intake and increased diabetes risk: experimental evidence for interplay between selenium and carbohydrate metabolism. J Clin Biochem Nutr.

[47] Alarcon MN, Vique CC (2008). Selenium in food and the human body: a review. Sci Total Environ.

[48] Juniper DT, Phipps RH, Morales ER, Bertin G (2009). Effects of dietary supplementation with selenium enriched yeast or sodium selenite on selenium tissue distribution and meat quality in lambs. Anim Feed Sci Tech.

[49] Schrauzer GN (2000). Selenomethionine: A review of its nutritional significance, metabolism, and toxicity. J Nutr.

[50] Thomson CD, Packer MA, Butler JA, Duffield AJ, O’Donaghue KL, Whanger PD (2001). Urinary selenium and iodine during pregnancy and lactation. J Trace Elem Med Biol.

[51] Bugel S, Larsen EH, Sloth JJ, Flytlie K, Overvad K, Steenberg LC, Moesgaard S (2008). Absorption, excretion, and retention of selenium from a high selenium yeast in men with a high intake of selenium. Food Nutr Res.

[52] Schobel HP (1998). Pregnancy-induced alterations in renal function. Kidney Blood Press Res.

[53] Cheung KL, Lafayette RA (2013). Renal physiology of pregnancy. Adv Chronic Kidney Dis.

[54] Constantine MM (2014). Physiologic and pharmacokinetic changes in pregnancy. Front Pharmacol.

[55] Parshad RK (1999). Effects of selenium toxicity on oestrous cyclicity, ovarian follicles, ovulation, and foetal survival in rats. Indian J Exp Biol.

[56] Zhang S, Lin H, Kong S, Wang S, Wang H, Wang H, Armant DR (2013). Physiological and molecular determinant of embryo implantation. Mol Aspects Med.

[57] Sekhon LH, Gupta S, Kim Y, Agarwal A (2010). Female infertility and abortion. Curr Womens Health Rev.

[58] Riesewijk A, Martin J, van Os R, Horcajadas JA, Polman J, Pellicer A, Mosselman S, Simon C (2003). Gene expression profiling of human endometrial receptivity on days LH + 2 versus LH + 7 by microarray technology. Mol Hum Reprod.

[59] Pizarro AT, Figueroa P, Brito J, Marin JC, Munroe DJ, Croxatto HB (2014). Endometrial gene expression reveals compromised progesterone signaling in women refractory to embryo implantation. Reprod Biol Endocrinol.

[60] Alonso MR, Blesa D, Simon C (1822). The genomics of the human endometrium. Biochim Biophys Acta.

[61] Kind K (2006). Diet around conception and during pregnancy – effects on fetal and neonatal outcomes. Reprod Biomed Online.

